# Harmonizing national growth references for multi-centre surveys, drug monitoring and international postmarketing surveillance

**DOI:** 10.1111/j.1651-2227.2011.02415.x

**Published:** 2012-01

**Authors:** M Hermanussen, C Aßmann, H Wöhling, M Zabransky

**Affiliations:** 1 AschauhofAltenhof, Germany; 2 National Educational Panel Study, Otto-Friedrich-Universität BambergBamberg, Germany; 3 Sandoz BiopharmaceuticalsHexal AG, Holzkirchen, Germany

**Keywords:** Growth reference, International growth and registry studies, Multi-centre survey, Postmarketing surveillance

## Abstract

**Aim:**

National European growth references differ. We aimed to convert (*harmonize*) currently used charts into a single unified interchangeable LMS format for each European nation.

**Methods:**

Nine currently used national European growth references from Belgium (2009), France (1979), Poland (2001), Sweden (2002), Switzerland (1989), the UK (1990), Italy (2006) and Germany (1979 and 1997) were harmonized and compared with the international WHO child growth standards and WHO growth reference data for 5–19 years.

**Results:**

European growth charts can be harmonized. The approach appears useful as height, and body mass index (BMI) is inappropriately represented by WHO references. European height references exhibit warping when plotted against the WHO reference. The French appears too short, the other Europeans too tall. Also, the BMI is not appropriately represented by the WHO references.

**Conclusions:**

*Harmonizing* references is a novel, convenient and cost-effective approach for converting historic and/or incomplete local or national growth reference charts into a unified interchangeable LMS format. *Harmonizing* facilitates producing growth references ‘on demand’, for limited regional purposes, for ethnically, socio-economically or politically defined minorities, but also for matching geographically different groups of children and adolescents for international growth and registry studies.

## Introduction

Human growth is a dynamic process that is usually visualized by plotting individual measurements on so-called growth charts. Growth charts are common tools in the paediatric practice (1). They are usually derived from large local or national cross-sectional surveys. National growth charts are available for most European and also for many non-European populations. Since several years, also *international growth reference charts* based upon global rather than local samples of children are being recommended (2). The rationale for such charts goes back to recommendations of a Working Group on infant growth established by the World Health Organization (WHO). The group emphasized the similarity in early childhood growth among diverse ethnic groups and suggested describing how children should grow rather than how children grow [(3), http://www.who.int/childgrowth/1_what.pdf]. International growth references appear particularly convenient when growth data from different ethnic or geographic sources need to be matched, e.g. in multi-centre growth surveys, international drug monitoring or postmarketing surveillance of growth hormone therapies. Yet, the apparent differences in growth between the various European populations still raise inconvenient questions about the validity of so-called international references.

Traditionally, body height, body weight and body mass index (BMI) are being described in absolute terms and plotted on national growth reference charts. These charts usually offer mean values, standard deviations (SD) and/or percentiles. Recently, this concept has further been developed. For clinical use, the LMS method has been recommended almost 25 years ago (4) using three parameters (L, M and S) to transform skewed data to normality. This method allows converting individual measurements y from the *measurement scale* (cm, kg, kg/m^2^) into the SD score (SDS) scale, a single unified format with z- or *SDS values* (5,6). Based on the principles of transformation techniques (7), the conversion in case of L ≠ 0 is achieved via

**Key notes**

- The making of most modern growth reference charts differs.
- We present a novel, cost-effective approach for converting (*harmonizing*) charts into a unified interchangeable parametric format.
- *Harmonizing* facilitates producing growth references ‘on demand’, for limited regional purposes, for ethnically, socio-economically or politically defined minorities, but also for matching geographically different groups of children and adolescents for international growth and registry studies.

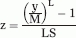
(1)

where z denotes a standard normal distributed random variable. The equation can be rewritten yielding


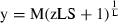


allowing the calculation of quantiles for y based on quantiles of z and given parameter values. Note that for L = 1, the parameters M and S correspond directly to the median/mean and the coefficient of variation of y. Note that for L = 0, the two above relationships render into z = [log (y/M)]/S and y = M exp [Sz]. Thus, given (estimated) parameters L, S and M from the population under consideration, SDS scores can be readily computed. A few modern growth references, e.g. the WHO (3) and UK reference curves (8), are already constructed according to the LMS method and allow converting measurements from *measurement scales* into *SDS scales*. Yet, the majority of the currently used European growth references still lack this option, which implies a major shortcoming when comparing child and adolescent growth between the various European nations.

## Material and methods

We selected nine currently used European national growth references from Belgium (9), France (10), Poland (11), Sweden (12,13), Switzerland (14), the UK (8,15,16), Italy (17) and two references from Germany [Reinken (18–20) and Hesse (21,22)]. Of these, only the UK reference provides full range LMS tables and allows complete conversion of height and BMI measurements into SDS for all ages. The Belgian reference provides LMS for height, but lacks BMI below 3, and the Italian reference lacks height and BMI below 2 years. The French, the Swiss and the German Reinken references provide full information on height, but lack BMI. The German Hesse and the Polish references provide information on height and 50th percentiles for BMI, and the Swedish reference provides information on mean values, skewness and kurtosis for height and BMI.

### Statistical approach

We tested whether these heterogeneous European growth references can be converted into one single interchangeable LMS format consisting of full range LMS tables for height and BMI from birth to maturity. Such a conversion would be an excellent practical solution – at least until *lege artis* assessed new national LMS references are available. For this goal, we developed a technique to amalgamate (*harmonize*) data that are already published and data that rely on estimates obtained from previous meta-analyses, e.g. (23). To achieve a harmonized representation of growth charts, we refer to the LMS methodology not implying that the LMS approach provides the best characterization for each population under consideration. In particular, for our purposes, we lack the feature of smoothing growth charts via use of spline functions underlying the parameters of LMS approach as given by (5). Merely, as an established technique, it shows of a feasible trade of between accuracy and tractability to achieve harmonization. We propose quite a heuristic technique for *harmonizing* formerly heterogeneous and incompatible references into one single unified and interchangeable SDS format, which is based on the publicly available heterogeneous information on European growth charts. Note that the LMS curve approach requires data on the individual level for each considered population. Unfortunately, for many populations, only few empirical moments are published; in particular, often even quantiles relevant for clinical purposes are not available. Thus, our approach aims at mapping available information (most often empirical moments like means and SD) into LMS parameters in order to provide harmonized growth charts providing all relevant information for clinical purposes. *Harmonizing* facilitates generating references ‘on demand’, particularly for limited regional purposes, for ethnically, socio-economically or politically defined minorities, but also for matching geographically different groups of children and adolescents for international growth and registry studies.

Our approach is related to the methods of moments principle allowing in general to base parameter estimation on empirical moments. Consider the case of L = 1. Then, the underlying Box Cox transformation in Eqn 1 implies the following moment conditions:


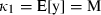



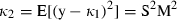


This moment conditions suggest the equations


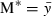


and


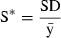


with 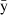 denoting the arithmetic mean and SD the standard deviation to be reasonable choices for the parameters given L = 1.

**Figure 1:**
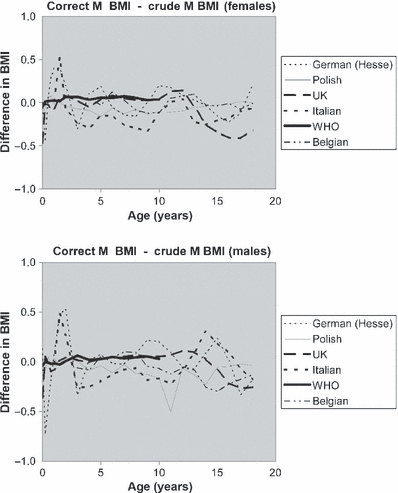
Correct M-values for body mass index (BMI) are defined by the individual weight/height^2^. As this information was absent in several studies, we instead created crude M-BMI by dividing mean weight by squared mean height. The differences between correct mean values and crude BMI appeared small in the German, Polish, UK, Italian, WHO and Belgian studies.

**Figure 2:**
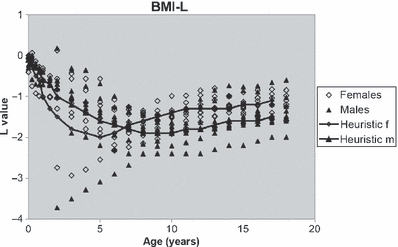
Published L-values of the Belgian (9), the UK (8,15,16), the Italian (17) and the WHO references, and studies published in the Netherlands (24), Germany (25), Sweden (26) and Brazil (27). The obvious differences in L are not explicable by particular features of the populations. Through lines indicate heuristic reasonable choices of L-values (Table 1).

This principle can be generalized to the situation L ≠ 0 implying moment conditions of the form κ_1_ = E[y] = f_1_(M, S, L), κ_2_ = E[(y − κ_1_)^2^] = f_2_(M, S, L) and κ_3_ = E[(y − κ_1_)^3^] = f_3_(M, S, L). These moments are given as integrals, e.g.


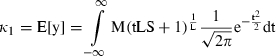


and have no closed form solution for general value of λ ≠ 0. Hence, simulation techniques are applied to solve the integrals and the corresponding non-linear equation system in turn. In case of information available on quantiles, the corresponding condition


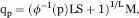
(2)

where p denotes the underlying probability threshold and ϕ^−1^ the inverse of the standard normal distribution, can be used to calculate reasonable values for the parameters. In particular, this relation reduces the complexity of the non-linear equation system, as it provides an explicit relationship between the three parameters of interest. Nevertheless, the transformation used between a standard normal random variable z and an observation y (Eqn 1) is not a one-to-one transformation that allows transforming any value from the interval −∞ to ∞ (range of z) towards only positive values (range of y), but depends on the restrictions z < −1/(LS) for L < 0 or z > −1/(LS) for L > 0, which was incorporated within the simulation of the above moments.The above principles are operationalized as follows. To ease the computational burden, we employed at most three empirical moments for identifying the LMS parameters. Further information present in additional reported empirical moments is neglected. The approach thus should be interpreted as a simplifying approximation in order to identify reasonable values for LMS parameters. Complications arise when direct information on three moments is missing. This is often the case for BMI. Given that information on two empirical moments are available only, we identify M and S based on a heuristic reasonable choice of L. L-values differ markedly between populations with widely overlapping 95% confidence intervals. The choice of L takes into consideration various L-values of Belgian (9), UK (8,15,16), Italian (17) and WHO references, and additional studies published in the Netherlands (24), Germany (25), Sweden (26) and Brazil (27). Arithmetic means obtained from these studies were considered a reasonable choice of L-values (Fig. 1, Table 1). Several growth studies only provide means of height and weight, but entirely lack BMI. As we are unable to exactly infer on LMS parameters, but rather have to approach a reasonable choice of these parameters, we estimated crude approximations of the mean BMI by the ratio of the involved means of weight and height (and squares thereof). Naturally, these approximations lack statistical foundation. As in some data sets, correct arithmetic means of BMI were available; we cross-checked the correct and the crude approximations and only found small differences that may be regarded negligible for clinical purposes (Fig. 2).

**Table 1:** Heuristic reasonable choices for BMI-L and BMI-S

Females			Males		
	
Age (years)	BMI-L	BMI-S	Age	BMI-L	BMI-S
0	0.001	0.074	0	0.001	0.085
0.25	−0.1	0.101	0.25	−0.1	0.077
0.5	−0.3	0.079	0.5	−0.2	0.079
0.75	−0.5	0.083	0.75	−0.3	0.091
1	−0.6	0.08	1	−0.4	0.086
1.5	−1	0.085	1.5	−0.6	0.087
2	−1.3	0.084	2	−0.7	0.084
3	−1.5	0.078	3	−1	0.071
4	−1.8	0.082	4	−1.2	0.072
5	−1.9	0.089	5	−1.4	0.075
6	−2	0.091	6	−1.6	0.081
7	−1.9	0.102	7	−1.7	0.092
8	−1.7	0.107	8	−1.8	0.098
9	−1.6	0.112	9	−1.9	0.102
10	−1.5	0.119	10	−1.9	0.108
11	−1.4	0.124	11	−1.9	0.112
12	−1.3	0.138	12	−1.8	0.117
13	−1.3	0.139	13	−1.8	0.119
14	−1.3	0.128	14	−1.7	0.122
15	−1.3	0.116	15	−1.6	0.117
16	−1.2	0.11	16	−1.6	0.115
17	−1.2	0.11	17	−1.6	0.115
18	−1.1	0.105	18	−1.5	0.117

BMI, body mass index.

### Weight

Weight depends on height (small persons tend to weigh less than tall ones), and as published information on weight is inconsistent, some references provide LMS for weight, others percentiles, others only mean values and SDs; the WHO references even lack information on weight beyond the ages of 10 years, we restricted the present work to height and BMI. This decision was supported by the fact that modern clinical decisions on nutritional status are anyway based on cut-off values for BMI and not on weight (28).

### Height

For clinical purposes, the parameter height can be considered normally distributed. Most growth references using LMS; therefore, set L = 1 at all ages. This was carried out in the references from Belgium, the UK and Italy. The other references provided mean values and SD for height that were converted into LMS tables with L = 1 for all ages. As the Italian references lacked information on height in infancy, we amended WHO height values for young ages.

### BMI

Weight and BMI are not normally distributed. LMS tables for BMI are present in the references from Belgium, the UK and Italy. The German (Hesse (21,22)) and the Polish (11) references provide 50th percentiles. The Swedish reference (12,13) provided arithmetic mean values for BMI, and the French (10), the Swiss (14) and the second German [Reinken (18–20)] references entirely lacked BMI. In these populations, we amended reasonable choices of L-values for BMI as described above.

Original S-values were only available in the Belgian, the UK and the Italian references. In the other references, we had to amend heuristic choices of reasonable S-values (Table 1, (29), Hermanussen, Meigen, unpublished). Percentiles can be used to estimate S-values according to Eqn 2. This was carried out in the Polish and in the Hesse references. The French, the Swedish, the Swiss and the Reinken references lacked percentiles. We therefore added a heuristic choice of S-values and separately compiled tables containing population-specific M-, L- and S-values for the Polish, the Hesse, the French, the Swedish, the Swiss and the Reinken references. Table 2 provides an example. This approach *harmonizes* original and reconstructed data into one single unified, interchangeable format consisting of complete LMS tables for the nine European growth references.

**Table 2:** An example for harmonizing: The German reference (Hesse) provides mean height (H M), standard deviation for height (H SD) [22] and percentiles for BMI [21]

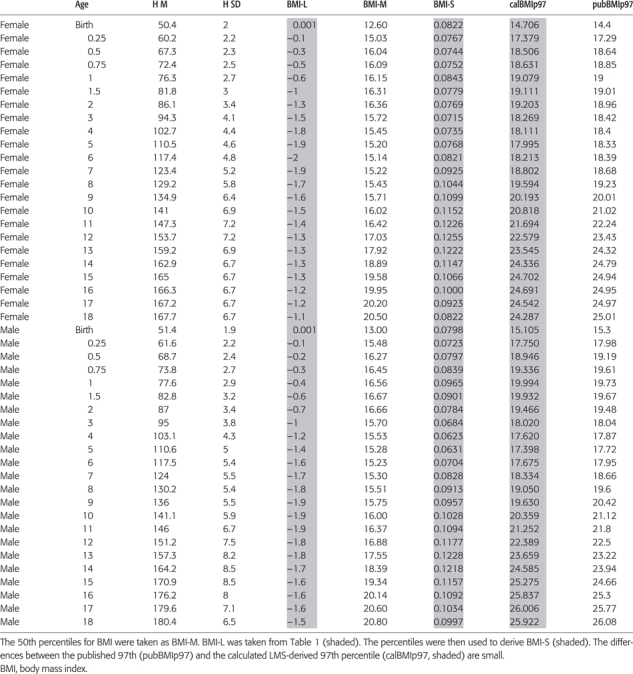

## Results

Table 2 exemplifies harmonized LMS for the German growth reference published by Hesse (21,22). Mean height (H M), SD for height (H SD) (22) and percentiles for BMI (21) were available. The 50th percentiles for BMI were taken as BMI-M. Heuristic BMI-L was taken from Table 1. The published percentiles for BMI were then used to amend BMI-S. The precision of this procedure can be assessed by comparing originally published and LMS-derived percentiles. Table 2 exemplifies the case of the 97th percentile: the originally published and the LMS-derived percentiles differed by 0.1 (SD = 0.4) BMI units.

The UK growth reference tables consisted of full range LMS tables (8,15,16) and did not need any amendments. Belgian BMI values needed amendment for ages below 3 years, and Italian height and BMI values were amended for ages below 2 years. In the other populations, BMI values have been amended at all ages, either based on correct [Poland, Sweden, Germany (Hesse)] or crude BMI-M [France, Switzerland, Germany (Reinken)].

**Figure 3:**
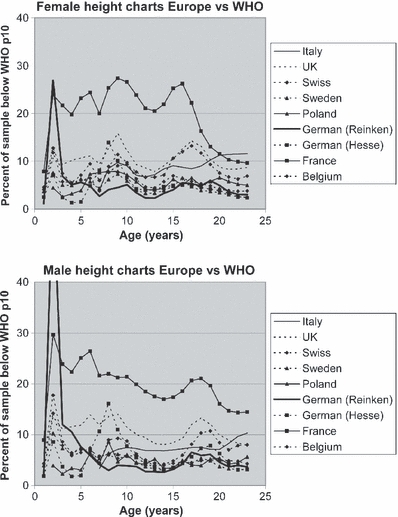
10th height percentiles of nine European growth references plotted on *WHO growth standard/references*. European 10th height percentiles markedly deviate from the WHO 10th height percentile. The percentiles were derived from LMS values. The figure clearly illustrates how inappropriately the European references are represented by the WHO references. The trough at 12–13 years indicates warping of the European percentiles against the WHO reference.

In many countries, the international WHO child growth standards and WHO growth reference data for 5–19 years are considered the gold standard for growth assessment. We therefore referred height of the nine European growth studies on WHO charts and tested the agreement between European and WHO references. Figure 3 exemplifies the 10th European height percentiles plotted on *WHO growth standard/references*. The figure illustrates that none of the European 10th height percentiles are appropriately represented by the WHO reference. Nearest to WHO is the UK reference, the French appears too short at all ages with some 25% of the young population below the 10th percentile of WHO height, other Europeans are too tall. All depicted European percentiles show a trough at 12–13 years indicating warping against the WHO reference.

**Figure 4:**
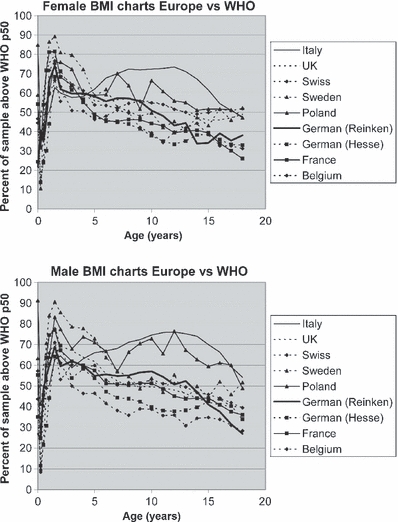
M-values for body mass index (BMI) of nine European growth references plotted on *WHO standard/references*. The figure clearly illustrates the inappropriateness also of WHO BMI references for the European populations regardless, whether the patterns of M-value were obtained from individual weight/height^2^ [Belgian (9), German (Hesse) (21,22), Polish (11), UK (8,15,16), Italian references (17)] or crude estimates. European infants are generally heavier than WHO standards suggest.

Similarly, incongruent results were obtained when plotting M-values for BMI on *WHO standard/references* (Fig. 4). Italians are particularly aberrant with more than 70% of the mid-pubertal adolescents surpassing mean WHO BMI. Regardless, whether the patterns of M-value for BMI were obtained from correct M-values, or 50th percentiles [Belgian (9), German (Hesse) (21,22), Polish (11), UK (8,15,16), Italian references (17)] or from crude M-values (France (10), Switzerland (14), Germany [Reinken (18–20)], the patterns appeared similar. European infants are in general heavier than WHO standards suggesting that the infant WHO BMI standards are not appropriate for European populations.

## Discussion

Child and adolescent growth have traditionally been viewed as an indicator of individual health [http://whqlibdoc.who.int/trs/WHO_TRS_854.pdf (30)] and society wellbeing (31). Yet, constructing and later actualizing empirical growth reference charts to identify individual health impairment is a demanding and expensive task. Even in Europe, little agreement exists in respect to which chart is the right chart to use (2).

LMS growth charts have been recommended to best fit modern clinical requirements (4–6), but international LMS charts do not appropriately represent ‘local growth’ of children of a certain geographic region (32). This dilemma has led to a confusing variety of modern and old, national and international growth charts. The incommensurability of growth charts severely complicates comparisons of child growth between different countries, and is particularly irksome in international health surveys, and in international drug monitoring and postmarketing surveillance programs. We thus pursued the idea of ‘upgrading’ frequently used traditional European growth references into modern full range (birth to maturity) LMS tables for height and BMI. In view of the promising efforts in constructing synthetic growth references for Lithuania (33) and several Russian populations (23), we started amalgamating, i.e. *harmonizing* available and heuristic data. This approach is simple in regard to height. As the parameter height is normally distributed, height tables that already consist of mean values and SD for height can immediately be converted into LMS (with L = 1). The approach is less trivial in regard to BMI.

LMS values for BMI were only available in the Belgian, the UK and Italian references. The German Hesse (21,22) and the Polish (11) references provided percentiles of BMI. The French (10), the Swiss (14) and the German Reinken (18–20) references entirely lacked BMI estimates. BMI is defined as weight/height^2^. Correct mean values for BMI should be obtained from all individual weight/height^2^ ratios. Yet, as this information was absent, we instead created crude mean values for BMI by dividing the mean values for weight by the squared mean values for height. Crude BMI differs from correct mean values for BMI, but the differences appeared to be close to zero and clinically negligible.

Given no further information, we compiled separate tables each containing M-, L- and S-values for the BMI. In this way, we *harmonized* the primarily incongruent European growth charts, resulting in strongly simplified, but interchangeable LMS tables for each country. Harmonizing growth charts appear to be a fascinating novel, convenient and cost-effective alternative to setting up *de-novo* growth studies. The technique facilitates producing references ‘on demand’, for limited regional purposes, for ethnically, socio-economically or politically defined minorities, but also for matching geographically different groups of children and adolescents for international growth and registry studies. Yet, the technique may raise problems, in that its simplicity may encourage people to neglect the true needs for properly raised original data. Nevertheless, we believe that if used carefully, a low-budget *harmonized* growth reference with its simple and unified SDS format provides significant advantages and may facilitate the investigation of worldwide variation in human growth.
